# Impaired Thymic Selection and Abnormal Antigen-Specific T Cell Responses in *Foxn1^Δ/Δ^* Mutant Mice

**DOI:** 10.1371/journal.pone.0015396

**Published:** 2010-11-04

**Authors:** Shiyun Xiao, Nancy R. Manley

**Affiliations:** Department of Genetics, Coverdell Center, University of Georgia, Athens, Georgia, United States of America; New York University, United States of America

## Abstract

**Background:**

*Foxn1^Δ/Δ^* mutant mice have a specific defect in thymic development, characterized by a block in TEC differentiation at an intermediate progenitor stage, and blocks in thymocyte development at both the DN1 and DP cell stages, resulting in the production of abnormally functioning T cells that develop from an atypical progenitor population. In the current study, we tested the effects of these defects on thymic selection.

**Methodology/Principal Findings:**

We used *Foxn1^Δ/Δ^*; DO11 Tg and *Foxn1^Δ/Δ^*; OT1 Tg mice as positive selection and *Foxn1^Δ/Δ^*; MHCII I-E mice as negative selection models. We also used an *in vivo* system of antigen-specific reactivity to test the function of peripheral T cells. Our data show that the capacity for positive and negative selection of both CD4 and CD8 SP thymocytes was reduced in *Foxn1^Δ/Δ^* mutants compared to *Foxn1^+/Δ^* control mice. These defects were associated with reduction of both MHC Class I and Class II expression, although the resulting peripheral T cells have a broad TCR Vβ repertoire. In this deficient thymic environment, immature CD4 and CD8 SP thymocytes emigrate from the thymus into the periphery. These T cells had an incompletely activated profile under stimulation of the TCR signal *in vitro*, and were either hypersensitive or hyporesponsive to antigen-specific stimulation *in vivo*. These cell-autonomous defects were compounded by the hypocellular peripheral environment caused by low thymic output.

**Conclusions/Significance:**

These data show that a primary defect in the thymic microenvironment can cause both direct defects in selection which can in turn cause indirect effects on the periphery, exacerbating functional defects in T cells.

## Introduction

The thymus is the primary site of T cell development. In the thymus, heterogeneous thymic epithelial cells (TECs) and other stromal cells including fibroblasts, dendritic cells, and macrophages constitute a 3-dimensional space that provides a unique microenvironment for the differentiation, proliferation, and maturation of T cells. During differentiation, T cells undergo VDJ recombination generating a diverse repertoire of TCRs. Thymic positive selection ensures the differentiation and maturation of thymocytes expressing a self-restricted TCR; negative selection then removes those thymoctyes that have dangerously high reactivity to self [1]. The resulting T cells migrate out of the thymus resulting in a diverse repertoire of T cells that are able to respond to foreign peptides while remaining tolerant to self-peptides. The positive selection of CD4 and CD8 T cells is restricted by MHCII and MHCI respectively. The development of TCRαβ CD4 T cells can be typically supported by MHCII expressing TECs, while CD8 T cells can be supported by MHCI signals from TECs and other stromal cells in thymus [2], [3], [4], [5], [6]. The thymic medulla has been shown to be largely responsible for negative selection [7], [8]. In the cortico-medullary junction and medulla, dendritic cells (DCs) play a major role in negative selection; medullary TECs have also been reported to be sufficient to drive this process [9].

The development, differentiation, and function of fetal TECs are controlled by the forkhead-class transcription factor Foxn1 [10], [11], [12], [13], [14]. Foxn1 is widely if not ubiquitously expressed in fetal TECs from a very early stage [13], [15]. In the absence of Foxn1 (i.e. in nude homozygotes), the thymic rudiment is formed, but lymphoid progenitor cells (LPCs) fail to populate it [12], [14], [16]. In a severe hypomorphic allele, *Foxn1^Δ^*, initial organogenesis appears normal, but later thymocyte-dependent TEC differentiation is blocked [2]. Thus, Foxn1 is required for both thymocyte-dependent and -independent TEC differentiation. In the postnatal thymus, *Foxn1* expression is distributed in both cortex and medulla [12], [17], [18]. We have recently reported that correct expression of *Foxn1* is also required to maintain the postnatal thymus, as postnatal reduction of Foxn1 expression leads to a phenotype resembling premature thymic involution [18]. Down regulation of Foxn1 has also been implicated in initiating thymic involution [19]. Thus, Foxn1 plays multiple crucial roles in the development and maintenance of TECs throughout the lifespan of the organism.

*Foxn1^Δ/Δ^* mice have a very small thymus with abnormal architecture lacking cortical and medullary domains, in which most TECs display an intermediate progenitor phenotype [10]. Thymocytes are specifically blocked at both the DN1 and DP differentiation stages, express constitutively low TCR, and very few SP thymocytes develop and immigrate into the periphery [10]. The resulting peripheral T cells have an atypical activated/memory phenotype with low homeostatic potential and impaired TCR signaling [20]. T regulatory (T_reg_) cells are generated but have reduced function, and T cells are hyper-responsive in both allo- and auto- MRL assays *in vitro* [21]. These abnormal T cells develop from CD117^−^ atypical progenitors without passing through the DN2 or DN3 stages, but directly develop into DN4 from DN1 cells in the postnatal thymus [20]. As these atypical progenitors are normally present in the wild-type thymus, these may represent a normal sub-population of T cells that may serve a specific as yet unknown function in the normal T cell repertoire. As these T cells also have constitutively low TCR expression, the question of whether this abnormal microenvironment is promoting normal positive and negative selection is also important to understanding the mechanism by which these T cells are generated.

In the current study, we investigated the processes of positive and negative selection in *Foxn1^Δ/Δ^* mutants. We show that TEC expression of both MHC Class I and II are reduced significantly, with MHC Class II expression affected more severely. We generated *Foxn1^Δ/Δ^* BALB/c (MHCII I-E molecule positive) mice, and crossed *Foxn1^Δ/Δ^* to the DO11.10 CD4 and OT1 CD8 restricted TCR transgenes. By focusing on the TCR Vβ usage and the phenotypes of CD4 and CD8 Tg cells in the thymus or periphery of *Foxn1^Δ/Δ^* mice, we analyzed the thymic negative and positive selection and assayed SP thymocyte post-selection maturation in the thymus. We also analyzed T cell responses to specific antigens *in vivo*. Our results show that the *Foxn1^Δ/Δ^* thymus did not generally affect the diversity of the TCR Vβ repertoire, although the frequencies of specific Vβ usage were highly variable in the mutants compared to controls. However, thymic negative selection was partially reduced and positive selection was dramatically reduced for CD4 T cells, but relatively normal for CD8 T cells. These T cells generated abnormal responses to antigen-specific stimulation *in vivo*. At least part of this abnormal response was secondary to the previously reported memory-like phenotype of T cells after lymphopenia-induced proliferation and reduced T_reg_ cell function in the *Foxn1^Δ/Δ^* periphery [20]. Thus, the peripheral T cell phenotype in the *Foxn1^Δ/Δ^* mutants is the result of both direct effects of abnormal selection within the thymus, compounded by secondary effects of low T cell output and a hypocellular peripheral environment.

## Results

### Peripheral T cells have broad but variable TCR Vβ usage in *Foxn1^Δ/Δ^* mutant mice

**Figure 1:**
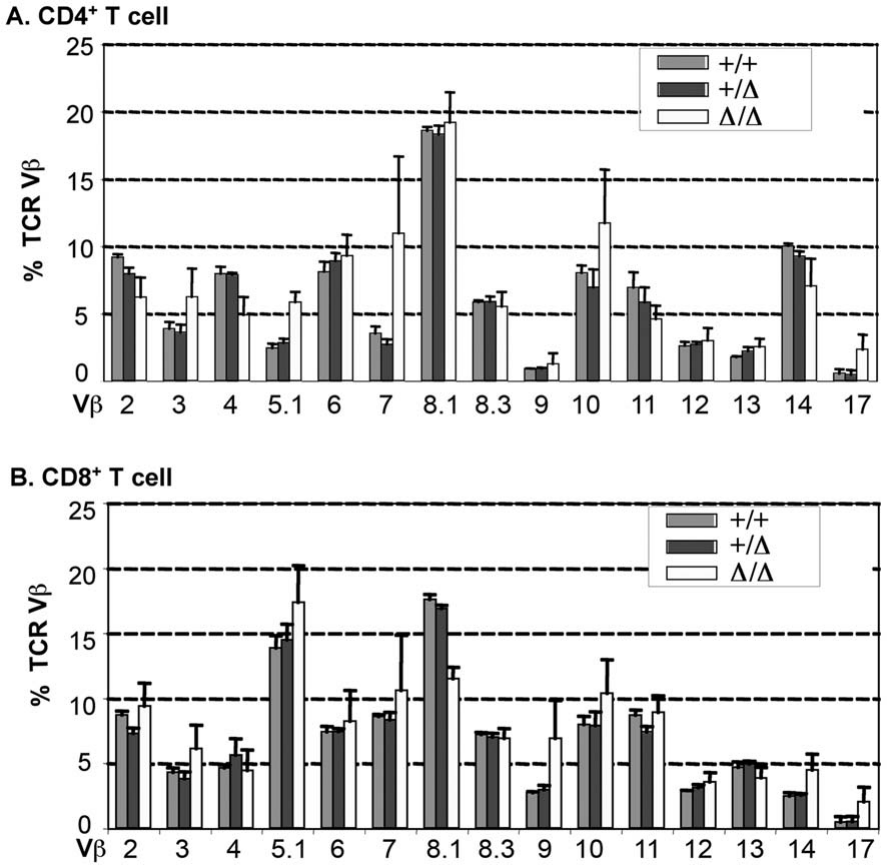
TCR Vβ usage in the peripheral T cells of *Foxn1^Δ/Δ^* mutant mice. The peripheral cells from spleen and LNs were screened with TCR Vβ usage. The result shown was average volume from at least 5 individual samples. A and B show the percentage of expression of TCR Vβs on CD4 and CD8 T cells.

Our previous data reported that T cells in *Foxn1* ^Δ/Δ^ thymus appear to develop from an atypical CD117^low/−^ progenitor that develops directly from DN1-DN4 stages, but did undergo TCR VDJ recombination [20]. This result raises the question of whether this atypical pathway generates a diverse TCR repertoire. Because the number of SP thymocytes in the *Foxn1* ^Δ/Δ^ thymus was too low to be analyzed directly for TCR Vβ usage, we assayed TCR Vβ expression on peripheral T cells (Fig. 1). We found that all TCR Vβ alleles were detected on both CD4 and CD8 *Foxn1* ^Δ/Δ^ T cells. Compared to TCRαβ^+^ T cells in control mice, there was no consistent difference in TCR Vβ expression between the *Foxn1* ^Δ/Δ^ mutant mice and controls. The one exception was TCR Vβ5, which was significantly over-represented in the *Foxn1* ^Δ/Δ^ CD4 T cells. However, a high variability of TCR Vβ expression was seen in *Foxn1* ^Δ/Δ^ individual mouse, compared to the consistent pattern of TCR Vβ expression on T cells from control mice. These results indicated that while all TCR Vβ alleles were utilized in the *Foxn1* ^Δ/Δ^ CD117^low/−^ progenitor T cells, either the relative frequency was variable, or the relative frequencies were skewed in the periphery as a consequence of low thymic output and differential expansion of specific Vβ clones in response to a hypocellular periphery.

### Thymic negative selection was partially reduced in the *Foxn1^Δ/Δ^* mutant mice

**Figure 2:**
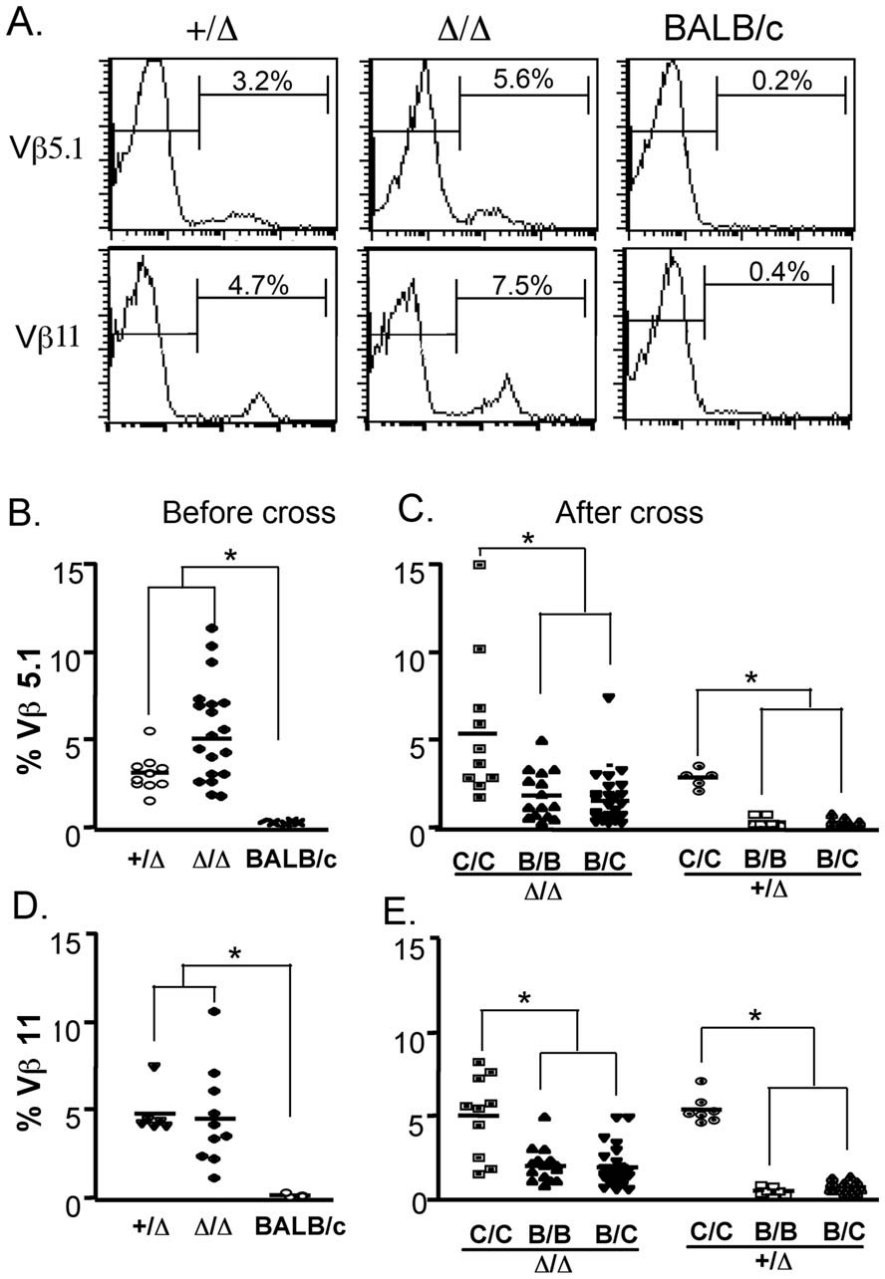
Thymic negative selection was partially reduced in the *Foxn1^Δ/Δ^* mutant mice. *Foxn1* ^Δ/Δ^/BALB/c mice were generated by backcrossing *Foxn1* ^Δ/Δ^ mice (H-2^b^, IE^-^) to BALB/c (H-2^d^, IE^+^) for more than 5 generations. Before and after backcrossing, the expressions of Vβ5.1 and Vβ11 were measured on CD4 T cells. A, histograms showing representative profiles of Vβ5.1 and Vβ11 expressions on gated CD4 T lymphocytes from *Foxn1^+/^* ^Δ^, *Foxn1* ^Δ/Δ^ and BALB/c mice. B and D, individual analyses of Vβ5.1 and Vβ11 expression on CD4 T cells from *Foxn1^+/^* ^Δ^, *Foxn1* ^Δ/Δ^ and BALB/c mice before backcrossing. C and E, individual analysis of the percentage of Vβ5.1 and Vβ11 from *Foxn1* ^Δ/Δ^ mice after backcrossing. C/C: C57BL6 background (IE^−^/IE^−^); B/B: BALB/c background (IE^+^/IE^+^); B/C: BALB/c/C57BL6 (IE^+^/IE^−^). (*, p<0.05).

Based on the expression of TCR Vβ, we designed an experiment to test thymic negative selection. Endogenous superantigen–mediated TCR Vβ T cell clonal deletion generally occurs in mice that express an endogenous super-antigen and the MHC-II I-E molecule [22]. The expression of Vβ5.1 and Vβ11 were clear on T cells in the *Foxn1^Δ/Δ^* and *Foxn1* ^+/*Δ*^ mice, both of which are maintained on the C57BL/6 background (Fig. 2A), because these mice failed to delete them due to lack of MHCII I-E molecules on TECs. In BALB/c mice, which express the MHCII I-E molecule, Vβ5.1 and Vβ11 bearing T cells are deleted (Fig. 2A, B, D) [22]. We therefore backcrossed the C57BL/6 I-E^−^ *Foxn1^Δ/Δ^* mice with BALB/c (I-E^+^) mice, generating BALB/c (I-E^+^) *Foxn1* ^Δ/Δ^ mice after more than 5 generations. On the BALB/c genetic background, the expression of Vβ5.1 and Vβ11 was greatly reduced in BALB/c *Foxn1^+/^* ^Δ^ control mice, with around 90% of Vβ5.1 and Vβ11 bearing cells deleted (Fig. 2B-E, Table 1). However only around 60% of Vβ5.1 and Vβ11 bearing cells were deleted in the *Foxn1* ^Δ/Δ^ mutant mice on this genetic background. These data showed that although the thymic environment was completely disorganized with lack of cortico-medullary junction and medulla, thymic negative selection did occur in the *Foxn1* ^Δ/Δ^ thymus, although with reduced efficiency. Because the percentage of MHC II^+^CD45^+^CD11c^+^ DCs was higher in total thymic stromal cells in the *Foxn1* ^Δ/Δ^ than in the *Foxn1^+/^* ^Δ^ mice (data not shown), these DCs may partially compensate for the mTEC deficiency in the *Foxn1* ^Δ/Δ^ thymus.

**Table 1:** Deletion of peripheral TCR Vβ5.1 and Vβ11 CD4^+^ T cell clones after backcrossing *Foxn1* ^Δ/Δ^ mice onto the BALB/c genetic background.

Cell clone	Vβ 5.1 T cell				Vβ 11 T cell			
Genotype	+/Δ		Δ/Δ		+/Δ		Δ/Δ	
Background	B/B	B/C	B/B	B/C	B/B	B/C	B/B	B/C
% Deletion	88.9	90.2	61.1	67.5	90.1	86.2	60	61.2

### Positive selection for CD4 T cells was greatly reduced in *Foxn1^Δ/Δ^* thymus

**Figure 3:**
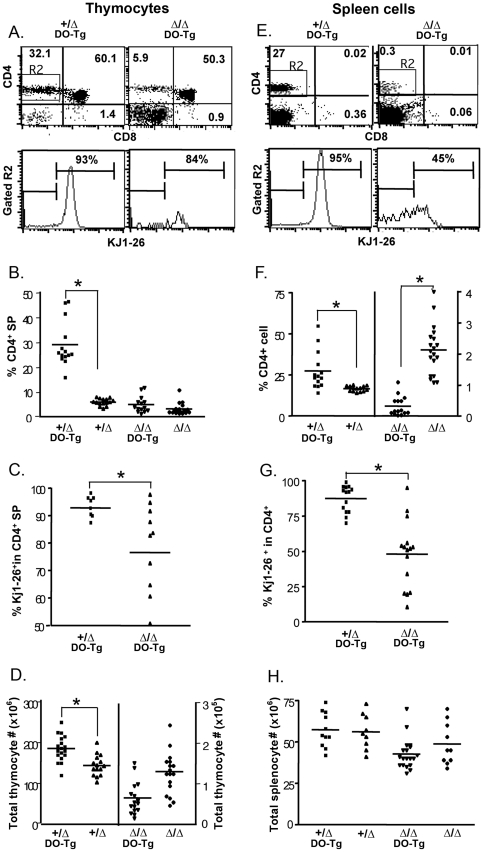
Positive selection for CD4 Tg T cells was greatly reduced in *Foxn1^Δ/Δ^*;DO11.10 Tg mice. *Foxn1* ^Δ/Δ^;DO11.10 and *Foxn1* ^Δ/Δ^;DO11.10 Tg mice were generated by mating *Foxn1* ^Δ/Δ^ BALB/c mice with DO11.10 CD4 TCR Tg mice, and then CD4 Tg T cells were analyzed in thymocyte (A, B, C and D) and splenocytes (E, F, G and H). A, representative profile of expression of CD4 and CD8 on thymocytes. Histograms showing expression of KJ1-26 on gated CD4 SP thymocytes. B, the percentage of CD4 SP thymocytes in individual DO11.10 Tg and non-Tg mice; C, individual analysis of percentage of KJ1-26^+^ cells on gated CD4 SP thymocytes; D, total number of thymic cells number from individual DO11.10 Tg and non-Tg mice. E, F, G and H show the same analyses performed on splenotytes. (*, p<0.05).

To measure the positive selection of CD4^+^ SP cells, we crossed the *Foxn1* ^Δ/Δ^;I-E^+^ mice with the DO11.10 CD4 restricted TCR transgenic mice (BALB/c background) to generate *Foxn1* ^Δ/Δ^;I-E^+^;DO11.10 Tg mice (called *Foxn1* ^Δ/Δ^;DO11.10 below) [23]. Compared to *Foxn1* ^+/Δ^ mice without the DO11.10 transgene, the percentage of CD4 SP thymocytes was increased 4-fold in *Foxn1* ^+/Δ^;DO11.10 mice (from 7.5% to 30.6%) (Fig. 3A, B), with around 93% of CD4^+^ SP cells being Kj1-26^+^ transgene-expressing T cells (Fig. 3C). The number of total thymocytes was also increased (Fig. 3D). In contrast, in *Foxn1* ^Δ/Δ^;DO11.10 mice the percentage of CD4^+^ SP thymocytes was not significantly increased compared to *Foxn1* ^Δ/Δ^ alone. Only 78% of CD4 cells were Kj1-26^+^, and the number of total thymocytes was not significantly changed (Fig. 3A, B, C, D). In peripheral T cells of *Foxn1^+/^* ^Δ^;DO11.10 control mice, the percentage of splenic CD4^+^ T cells was increased 1.7 fold compared to the non-transgenic mice, with 95% of them Kj1-26^+^ . However, in the *Foxn1* ^Δ/Δ^;I-E^+^;DO11.10 mice, the percentage of splenic CD4 T cell dropped dramatically down over 6 fold, and only 47% were Kj1-26^+^ cells (Fig. 3E, F, G, H). These results indicated that the capability for positive selection of the DO11.10 transgenic TCR was reduced in the *Foxn1* ^Δ/Δ^ mice. This phenotype showed high variability based on the wide range in percentage of Kj1-26^+^ CD4 T cells in the *Foxn1* ^Δ/Δ^;DO11.10 mice. In addition, we found a low percentage of Kj1-26^+^ CD4 T cells in the periphery compared to that seen in the thymus of *Foxn1* ^Δ/Δ^;DO11.10 mice, suggesting that the transgenic CD4^+^ cells might not survive well in the periphery of the *Foxn1* ^Δ/Δ^;DO11.10 mice.

### Positive selection for CD8 T cells was partially reduced in *Foxn1^Δ/Δ^* thymus

**Figure 4:**
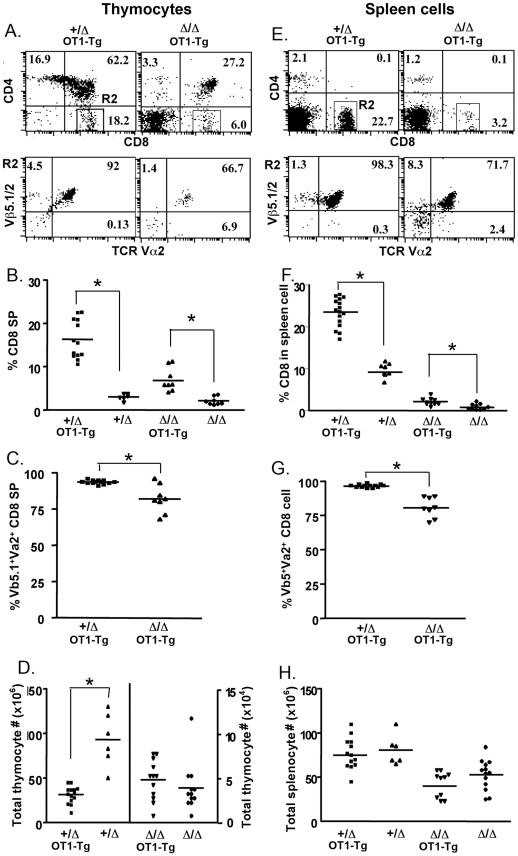
Positive selection for CD8 Tg T cells was partially reduced in *Foxn1^Δ/Δ^* mice. *Foxn1* ^Δ/Δ^;OT1 and *Foxn1* ^Δ*/*Δ^;OT1 Tg mice were generated by mating *Foxn1* ^Δ/Δ^ mice with OT1 CD8 TCR Tg mice, and the CD8 Tg cells were analyzed in thymocytes (A, B, C and D) and splenocytes (E, F, G and H). A, representative profile of CD4 and CD8 expression on thymocytes (top panel), and expression of Vα2 and Vβ5 on gated CD8 SP thymocytes (bottom panel). B, percentage of CD8 SP thymocytes in individual OT1 Tg and non-Tg mice; C, individual analysis of the percentage of Vα2^+^ and Vβ5^+^ cell on gated CD8 SP thymocytes; D, total number of thymic cells from individual OT1 Tg and non-Tg mice. E, F, G and H show the same analyses performed on splenotytes. (*, p<0.05).

To measure the positive selection of CD8^+^ T cells, we crossed the *Foxn1* ^Δ/Δ^ mice with OT1 TCR transgenic mice, in which thymocytes are restricted to development of CD8^+^ T cells. Tg CD8 T cells were identified by expression of TCR Vβ5 and Vα2. In the thymus of *Foxn1^+/^* ^Δ^;OT1 mice, the percentage of CD8^+^ SP cells was increased 5.4 fold (from 3% to 16.2%) compared to *Foxn1^+/^* ^Δ^ mice without the transgene, and 94% of CD8^+^ SP thymocytes were transgene positive. In *Foxn1* ^Δ/Δ^;OT1 mice, the percentage of CD8 SP thymocyte was increased 3 fold (from 2.1% to 6.2%), and 81.9% CD8 SP thymocytes were CD8 Tg cells (Fig. 4A, B, C). Compared to non-transgenic mice, the total thymic cell number was reduced in the *Foxn1^+/^* ^Δ^;OT1 control mice, but was unchanged in *Foxn1* ^Δ/Δ^;OT1 mice (Fig. 4D). For peripheral T cell analysis, the results were similar to thymus, except there was no significant difference in total cell number between mice of different genotypes (Fig. 4E, F, G, H). These results indicated that *Foxn1* ^Δ/Δ^;OT1 mice had a reduced capability for producing CD8^+^ OT1 transgenic T cells compared to the *Foxn1^+/^* ^Δ^;OT1 control mice, although to a lesser extent than was seen for positive selection of CD4+ T cells.

### Reduced MHC expression in *Foxn1^Δ/Δ^* TECs

**Figure 5:**
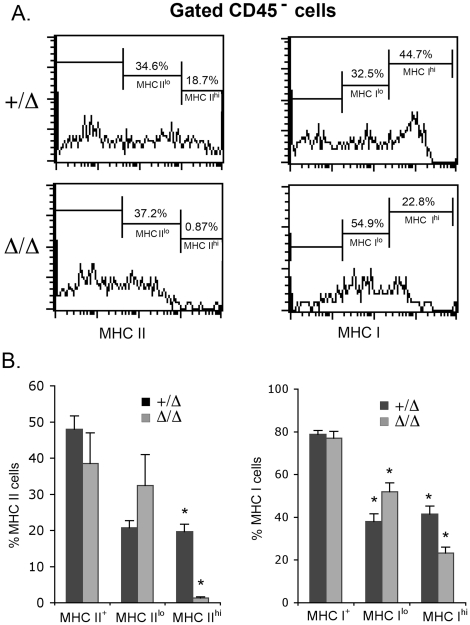
Changes in MHCII and MHC I expression on *Foxn1^Δ/Δ^* TECs. A, histograms showing representative profile of MHC II (left) and MHC I (right) expression on gated CD45^−^ thymic stromal cells from *Foxn1^+/^* ^Δ^ and *Foxn1* ^Δ/Δ^ mutant C57BL/6J mice. B, the percentage of MHC II^+^ and MHC II^hi^ cells (left), MHC I^+^ and MHC I^hi^ cells (right) in gated CD45^−^ thymic stromal cells from *Foxn1^+/^* ^Δ^ and *Foxn1* ^Δ/Δ^ mutant C57BL/6J mice. (*, p<0.05).

The evidence for decreased positive and negative selection in the *Foxn1* ^Δ*/*Δ^ mutant thymus combined with our previous data showing serious deficits in TEC differentiation and cortico-medullary organization in these mutants suggested that MHC expression could well be affected. MHC II^hi^ stromal cells were almost completely absent from the *Foxn1* ^Δ/Δ^ mutant thymus, and the percentage of stromal cells expressing intermediate levels of MHC II was variable in the mutants, although not significantly different (Fig. 5). MHC I^hi^ cells were also reduced significantly, although not as dramatically, while MHC I^lo^ were increased proportionately. The difference in magnitude of the effects on Class I and Class II MHC was consistent with the more severe effect on the positive selection of CD4^+^ SP cells in the *Foxn1* ^Δ/Δ^ mutants.

### Maturation of SP thymocytes was delayed in *Foxn1^Δ/Δ^* mice and immature SP thymocytes migrate into the periphery

In the postnatal thymus, SP thymocytes stay in the thymic medulla after positive selection for 3 to 5 days for further maturation [24], [25]. We previously reported that the thymic architecture was completely disorganized in the *Foxn1* ^Δ/Δ^ mice, with lack of cortico-medullary junction and medullary organization, as well as decreased TEC differentiation [10], although SP thymocytes do leave the thymus and colonize the periphery [20]. This raises the question of whether and how SP thymocytes undergo maturation in this deficient environment. To address this issue, we assayed expression of the maturation markers HSA and Qa2 on adult peripheral T cells. As we have previously shown that SP thymocytes in the *Foxn1* ^Δ/Δ^ thymus are contaminated with recirculated peripheral T cells reentering the very hypocellular *Foxn1* ^Δ/Δ^ thymus [10], [20], we tracked the expression of these markers on thymocytes from newborn through 28 days postnatal, and compared them to *Foxn1^+/^* ^Δ^ controls.

**Figure 6:**
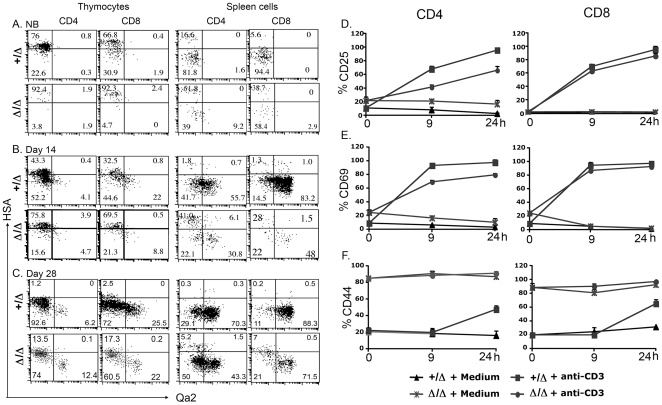
SP thymocytes and splenocytes show immature surface phenotypes and incomplete activation upon stimulation with anti-CD3. A, expression of HSA and Qa2 on thymocytes (left panel) and splenoytes (right panel) derived from NB, day 14 and day 28 *Foxn1^+/^* ^Δ^ and *Foxn1* ^Δ/Δ^ mice. D, E and F, expression of CD25, CD69, CD44 on peripheral CD4 (left) and CD8 (right) T cells after stimulation with anti-CD3 for 9 and 24 hours in vitro. The results shown were the average from two individual experiments.

In control mice at the newborn stage, most CD4 and CD8 SP thymocytes are HSA^hi^, while in the spleen the vast majority of T cells have down regulated HSA, and there is essentially no Qa2 expression in either population (Fig. 6A). By Day 14, over half of SP thymocytes have down regulated HSA, and a few also have low levels of Qa2. In contrast, over 50% of CD4 and over 80% of CD8 splenic T cells are Qa2^+^, and virtually all are HSA negative (Fig. 6B). This trend continues in the thymus such that 4 weeks of age most thymic SP cells are HSA^lo^Qa2^−^, while most splenic T cells are HSA^−^Qa2^+^ (Fig. 6C).

In the *Foxn1* ^Δ/Δ^ mutants at all ages examined, both SP thymocytes and splenic T cells showed differences in their HSA and Qa2 profiles compared to controls. At the newborn stage, over 90% of thymocytes were HSA^hi^, while over 50% of CD4 and 35% of CD8 splenic T cells also showed this phenotype (Fig. 6A). At Day 14, about twice as many SP thymocytes remained HSA^hi^ in the mutants compared to controls, although the frequency of HSA^lo^Qa2^+^ thymocytes was similar. However, in the spleen the effect was much greater, with 40% of CD4 and 30% of CD8 T cells remaining HSA^hi^, and the percentage of Qa2^+^ cells was roughly half that of controls (Fig. 6B). Even at Day 28, roughly 15% of SP thymocytes and 5–7% of splenic T cells retained high HSA levels, ten-fold that of control mice, and fewer splenic T cells were Qa2^+^ (Fig. 6C). Thus, down-regulation of HSA and up-regulation of Qa2 on both CD4 and CD8 SP thymocytes and on peripheral T cells were delayed in *Foxn1* ^Δ/Δ^ mice.

One explanation for the presence of HSA^hi^ peripheral T cells is that some immature *Foxn1* ^Δ/Δ^ SP thymocytes might migrate out of the thymus. Consisted with this possibility, we found that CD4 peripheral T cells from the *Foxn1* ^Δ/Δ^ mice could not be completely activated. Around 20% of CD4 cells from *Foxn1* ^Δ/Δ^ mice could not up-regulate the expression of CD25 or CD69 after stimulation with anti-CD3 within 24 hours *in vitro* (Fig. 6D). Almost all *Foxn1^+/^* ^Δ^ CD4 cells were activated with the same stimulation. In contrast, *Foxn1* ^Δ/Δ^ CD8 SP cells were more similar to the profile of the *Foxn1^+/^* ^Δ^ CD8 cells, with almost complete activation. Consistent with our previous results, *Foxn1* ^Δ/Δ^ T cells had constitutively high expression of CD44, so this was not a reliable marker for activation status [21](Fig. 6D).

### *Foxn1^Δ/Δ^* peripheral CD4, CD8 T cells have different responses to SEA super-antigen *in vivo*

**Figure 7:**
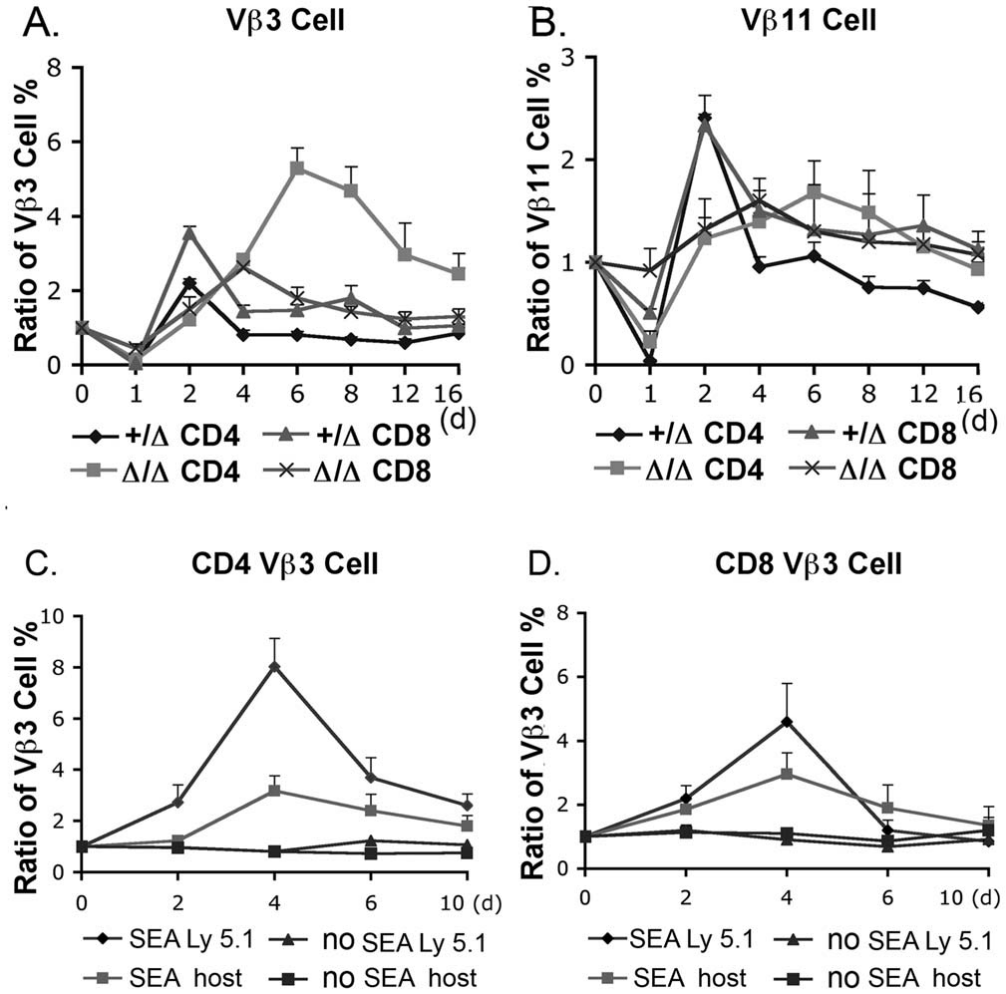
*Foxn1^Δ/Δ^* peripheral T cells have abnormal responses to SEA *in vivo*. A and B, showing kinetic response of Vβ3 and Vβ11 T cells to SEA *in vivo*. *Foxn1^+/^* ^Δ^ and *Foxn1* ^Δ/Δ^ mice were retro-orbitally injected with SEA 1 µg per mouse, and the percentages of Vβ3 and Vβ11 were measured in peripheral blood T cells on the indicated day. Y values show the ratio of percentages of Vβ3^+^ and Vβ11^+^ cell in individual day relative to day 0 (before injection of SEA). C and D, the response of transferred Ly5.1^+^ cells to SEA in *Foxn1* ^Δ/Δ^ mice. Sorted Ly5.1 T cells at 4×10^6^ per mouse were transferred into *Foxn1* ^Δ/Δ^ mice. One day later, the percentage of Vβ3 was measured as day 0 and then SEA 1 µg per mouse was injected. The percentage of Vβ3 was continuously measured on the indicated days after injection of SEA and shown as the ratio of the percentage relative to day 0. The results shown were the average from two or three individual experiments.

We next assayed how these T cells responded to specific antigen in the periphery. We first tested the response of TCR Vβ T cell clones to the super-antigen SEA *in vivo*. Before and after exposure to SEA, the expression of Vβ3 and Vβ11 were measured in individual mice. Because the frequency of Vβ3 and Vβ11 bearing cells in individual *Foxn1* ^Δ/Δ^ mice was quite variable, the percentage change in Vβ3/11 bearing cells in individual mice was shown by the ratio of before and after injection. As expected, the percentage of Vβ3/11 bearing CD4 and CD8 T cells increased 2–4 fold after exposure to SEA at day 2 post-injection in the *Foxn1^+/^* ^Δ^ mice, and then went down rapidly to the same or even below the level seen before exposure (Fig. 7), similar to a previous report [20]. However in the *Foxn1* ^Δ/Δ^ mice, the Vβ3-bearing CD4 T cells showed a delayed but a stronger and longer lasting response, with a 5-fold increase in percentage at day 6 after SEA exposure, and then a slow decline. CD8 T cells also had a delayed response although it was weaker than CD4 T cells, and even weaker than *Foxn1^+/^* ^Δ^ CD8 T cells (Fig. 7A). Both *Foxn1* ^Δ/Δ^ Vβ11 bearing CD4 and CD8 cells had a delayed and weaker response to SEA compared to the same cells in the *Foxn1^+/^* ^Δ^ mice (Fig. 7B). These data indicated that different *Foxn1* ^Δ/Δ^ T cell clones have different responses to the same antigen in vivo. Thus, some clones might increase and some clones might be reduced in response to the same antigen.

The thymus defects in the *Foxn1* ^Δ/Δ^ mice result in very low SP cell production and decreased T cell output from the thymus, causing a hypocellular peripheral environment [10], [20]. The hypocellular periphery contributes to the peripheral T cell phenotypes by promoting an activated/memory phenotype in most T cells [20]. To distinguish whether the abnormal response of *Foxn1* ^Δ/Δ^ T cells was directly due to the developmental defect in the thymus or the environmental defect in periphery, 4×10^6^ sorted Ly5.1 wild-type peripheral T cells per mouse were retro-orbitally transferred into *Foxn1* ^Δ/Δ^ adult mice. One day later, 1 µg SEA per mouse was injected. The percentages of Vβ3 bearing T cells of both host and donator were measured before and after injection of SEA and shown as the ratio of percentage (Fig. 7B). After 2 days of exposure to SEA, the percentage of transferred Ly5.1 Vβ3 bearing wild-type CD4 cells was increased more than 2 fold, which reached a peak of 8 fold at day 4 and then reduced. A similar result was showed in Ly5.1 wild-type CD8 cells. The percentages of transferred Vβ3 bearing CD4 and CD8 clones did not increase in the absence of SEA injection, indicating that the increase in transferred Vβ3 bearing CD4 and CD8 clones was not due to homeostatic proliferation. These results suggest that the hypocellular peripheral environment shapes the response of T cells to specific antigen. The host Vβ3 bearing CD4 cells had a weaker response to SEA than transferred Ly5.1 wild-type cells, although the magnitude of their response was similar to that seen in panels A and B. This suggests that in direct comparison to wild-type cells, the *Foxn1* ^Δ/Δ^ peripheral T cells have a weaker response to specific antigen. It is also possible that the low expression of surface TCR on *Foxn1* ^Δ/Δ^ T cells resulted in a reduced ability to compete for limited antigen stimulation in the same mouse.

### *Foxn1^Δ/Δ^*;OT1 TCR transgenic CD8 cells have a high sensitivity response to OVA protein

**Figure 8:**
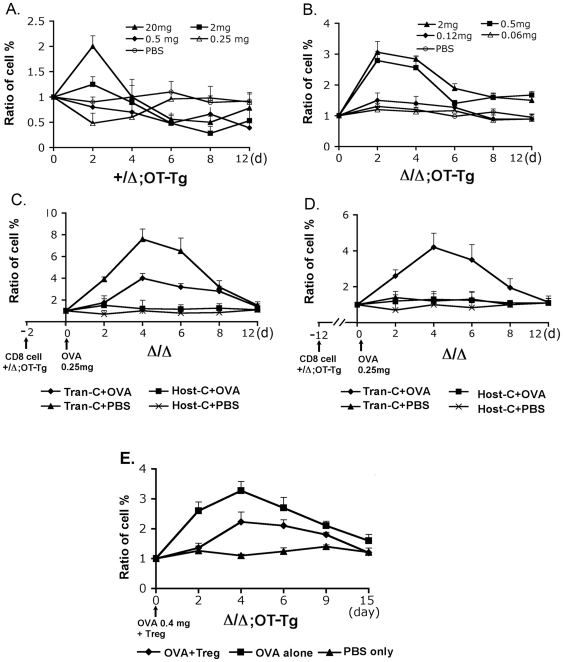
*Foxn1^Δ/Δ^*;OT1 Tg CD8 cells have a high sensitivity response to OVA protein in *Foxn1^Δ/Δ^*;OT1 Tg mice. All results are shown as a ratio of the percentage of OT1 Tg CD8 T cells on the indicated day relative to day 0. A and B, kinetic response of OT1 Tg CD8 T cell to serial doses of OVA. The *Foxn1^+/^* ^Δ^;OT1 Tg (+/Δ;OT-Tg) and *Foxn1* ^Δ/Δ^;OT1 Tg (Δ/Δ;OT-Tg) mice received a single retro-orbital injection of OVA from 0.06 to 20 mg, and CD8 Tg T cells in individual mice were analyzed at the indicated day. C and D, response of transferred *Foxn1^+/^* ^Δ^;OT1 Tg CD8 T cells to OVA in *Foxn1* ^Δ/Δ^ mice. 2×10^6^ sorted *Foxn1^+/^* ^Δ^;OT1 Tg CD8 T cells were transferred into *Foxn1* ^Δ/Δ^ mice. 1 day (C) and 10 days (D) later, the percentage of OT1 Tg CD8 cell was measured as day 0 and then OVA 250 µg per mouse was injected. The percentage of OT1 Tg CD8 T cells was continuously measured at the indicated day after OVA injection. Results shown were the average volume from two or three individual experiments. E, the hypersensitive response of *Foxn1* ^Δ/Δ^;OT1 Tg CD8 T cells was partially suppressed by the transferred CD4^+^CD25^+^ regulatory T cells. Sorted CD4^+^CD25^+^ T cells were retro-orbitally transferred into *Foxn1* ^Δ/Δ^;OT1 mice combining with OVA 400 µg per mouse. The results shown were the average volume from two individual experiments.

To further investigate the abnormal response of the *Foxn1* ^Δ/Δ^ T cell, the *Foxn1* ^Δ/Δ^;OT1 Tg (marked as Δ/Δ;OT-Tg in Fig. 8) mice were used to analyze the response of CD8 cells to OVA protein. Serial doses of OVA protein were injected into *Foxn1* ^Δ/Δ^;OT1 Tg and *Foxn1* ^+/Δ^;OT1 Tg (marked as +/Δ;OT-Tg in Fig. 8) mice. In all experiments, the day of OVA injection was set as day 0. The percentage of CD8^+^;OT1 Tg cells was measured before and after injection of OVA, and the change of percentage of CD8^+^;OT1 Tg cell was shown as a ratio. In *Foxn1* ^+/Δ^;OT1 Tg control mice, CD8^+^;OT1 Tg T cells expanded at day 2 after exposure to relative high doses of OVA. Between day 2 and day 4 after injection of OVA, the percentage of CD8^+^;OT1 Tg cells dropped quickly, and decreased to below the day 0 level after 4 days. The response was dose dependent. For example, 20 mg OVA gave a 2-fold increase of Tg CD8 population in percentage, and 2 mg OVA gave a 1.3 fold increase. Doses of OVA below 2 mg caused a decrease in the percentage of CD8^+^;OT1 Tg cells (Fig. 8A), as previously reported [26], [27]. In the *Foxn1* ^Δ/Δ^;OT1 Tg mice, a hypersensitive response was seen in the CD8^+^;OT1 Tg population. Doses of 0.5 mg were enough to drive a dramatic expansion of CD8^+^;OT1 Tg cells at day 2 to a level higher than that seen with the 20 mg dose in *Foxn1* ^+/Δ^;OT1 Tg control mice. This response lasted 2 days longer than the response in *Foxn1* ^+/Δ^;OT1 Tg control mice, then fell to the level of day 0 at day 6. At no dose did the CD8^+^;OT1 Tg population significantly decrease to below the level of day 0 in *Foxn1* ^Δ/Δ^ mice (Fig. 8B). Thus, the *Foxn1* ^Δ/Δ^;OT1 Tg T cells had a hypersensitive proliferative response to low doses of antigen, and never responded to antigen by with clonal deletion.

We have previously shown that peripheral T cells in the *Foxn1* ^Δ/Δ^ mice have an activated/memory phenotype due to lymphopenia-induced proliferation (LIP) [20]. To test whether the hypersensitive response of *Foxn1* ^Δ/Δ^;OT1 Tg CD8^+^cells was due in part to exposure to the lymphopenic environment of the *Foxn1* ^Δ/Δ^ mice, rather than to differences in intrathymic development, we transferred CD8^+^;OT1 Tg cells derived from *Foxn1* ^+/Δ^;OT1 Tg mice into *Foxn1* ^Δ/Δ^ mice that did not carry the OT1 transgene (‘host’ in Fig. 8C–E). Two days later (Day 0) we injected a low dose of OVA at 0.25 mg per mouse. As above, the percentage of CD8^+^;OT1 Tg cells was measured before and after injection of OVA, and the change of percentage of CD8^+^;OT1 Tg cells was shown as a ratio. As expected, host T cells showed no change either in the presence or absence of OVA over a 12-day period (Fig. 8C). The percentage of transferred CD8^+^;OT1 Tg cells increased at day 2 after injection of OVA, and then reached a peak to 8 fold higher than the level of day 0, consistent with an LIP response in the hypocellular *Foxn1* ^Δ/Δ^ peripheral environment [20]. In recipient mice that were given injection of OVA, the percentage of CD8^+^;OT1 Tg cells increased more slowly and reached a lower peak (3 fold increase) than with cell transfer alone. Our interpretation of this result is that the expansion of CD8^+^;OT1 Tg cells was driven by LIP, and that the low dose of 0.25 mg OVA induced some level of clonal deletion in the CD8^+^;OT1 Tg, as it did in the *Foxn1* ^+/Δ^;OT1 Tg mice (Fig. 8A). If the OVA had further stimulated the LIP effect, the percentage of CD8^+^;OT1 Tg cell should have been higher than the percentage in the cell transfer control group (Fig. 8C). Thus, with OVA challenge immediately after cell transfer, the transferred CD8^+^;OT1 Tg cells underwent clonal deletion in response to the i.v. injection of OVA.

The possibility that we were inducing simultaneous LIP and clonal deletion in the above experiment raised the issue of whether we could separate the two responses. Another experiment was performed which OVA was injected 12 days after CD8^+^;OT1 Tg cells were transferred. At this time, all the transferred CD8^+^;OT1 Tg cells had already undergone LIP, and displayed a proliferating status and were CD44^hi^, but the percentage had returned to a level similar to day 0 (Fig. 8C and data not shown). This situation was therefore more similar to that of the host T cells in the *Foxn1* ^Δ/Δ^ mice [20]. In controls that received cells only, the percentage of CD8^+^;OT1 Tg cells did not change over the additional 12-day period without OVA injection (Figure 8D). In contrast, after injection of OVA the percentage of CD8^+^;OT1 Tg cells increased 2 days later, with a overall profile similar to that seen with injection of OVA soon after cell transfer (Figure 8C), rather than undergoing deletion as seen in the *Foxn1* ^+/Δ^;OT1 Tg mice. This result indicated that he response to OVA was dictated not by the thymic environment in which they developed, but by the peripheral environment in which they were exposed to OVA. We interpret this result to mean that exposure to the lymphopenic peripheral environment of the *Foxn1* ^Δ/Δ^ mice and subsequent LIP-induced development of a CD44^hi^ memory like phenotype conferred to the transferred cells a hypersensitive response to specific antigen.

### CD4^+^CD25^+^ regulatory T cells can partially suppress the response of the *Foxn1^Δ/Δ^*;OT1 Tg CD8^+^ cells to OVA *in vivo*

One possibility for the high response of CD8^+^;OT1 Tg cells in the *Foxn1* ^Δ/Δ^ peripheral environment to OVA was the lack of normal regulatory T cell (T_reg_) function in *Foxn1* ^Δ/Δ^ mice [20]. To address this issue, sorted CD4^+^CD25^+^ Treg cells from the *Foxn1^+/^* ^Δ^ control mice were transferred into the *Foxn1* ^Δ/Δ^;OT1 Tg mice together with i.v. injection of OVA at 0.4 mg per mouse, an compared to injection of OVA or PBS alone. In the OVA only group, the percentage of CD8^+^;OT1 Tg cells increased as expected (compare to Figure 8E with 8B). In the OVA plus Treg group, the percentages of Tg CD8 cells were decreased comparing to the percentage in OVA non-Treg cells group (Fig. 8E), indicating that the co-injected Treg cells could suppress the proliferative response to antigen. These results indicated that the reduced function of CD4^+^CD25^+^ Treg cells in *Foxn1* ^Δ/Δ^ might contribute to the high response of CD8^+^;OT1 Tg cells to OVA in the *Foxn1* ^Δ/Δ^ peripheral environment.

## Discussion

Our previous work has shown that the thymic microenvironmental defects in the *Foxn1^Δ/Δ^* mouse result in a variety of secondary effects on thymocyte development and peripheral T cell function [10], [20], [21]. The primary result from these previous studies was that T cells in the *Foxn1* ^Δ/Δ^ thymus developed from kit negative progenitor cells via an atypical differentiation pathway. Our current data further explore the mechanisms by which this defective microenvironment influences thymocyte development, specifically positive and negative selection, and how these defects impact T cell function. We show that these atypical T cells have surprisingly normal Vβ usage, but significant defects in positive and negative selection, especially for CD4^+^ SP cells. Furthermore, immature SP cells were able to exit the thymus prematurely. These defects were exacerbated by peripheral lymphopenia caused by the low thymic output, and the poor function of T_reg_ cells generated in the *Foxn1* ^Δ/Δ^ mice. Thus, peripheral T cell phenotypes were due to both primary defects in the thymic microenvironment and secondary conditions in the peripheral environment.

Canonical TCRαβ progenitors perform VDJ recombination randomly after commitment, but positive selection for different Vβ may need continuous development of a variety of TECs [5]. In spite of the severe defects in *Foxn1* ^Δ/Δ^ TEC differentiation and the lack of organized thymic compartments, we found that Vβ usage on peripheral T cells was largely similar to control mice. This result suggested that these atypical progenitors had relatively normal VDJ recombination of TCR Vβ, that the severe TEC defect in the *Foxn1^Δ/Δ^* thymus did not significantly affect the ability to support the process of TCRαβ commitment, and that there was no selective loss of specific Vβ bearing clones during positive selection. However, the peripheral Vβ repertoire was highly variable between mice. This variability could reflect variability in the differentiation and/or organization of the thymic environment between different mutant mice. Alternatively, T cells with higher affinity to MHC may undergo selective expansion via homeostatic induced proliferation in the *Foxn1* ^Δ/Δ^ lymphopenic periphery, where MHCII expression should be normal. In the case of TCR Vβ5, this allele was reported to advantageously expand in inflammatory bowl disease (IBD) [28], [29]. As our previous data also showed that some *Foxn1* ^Δ/Δ^ mice showed autoimmune disease symptoms including prolapse [20], the increased TCR Vβ5 expression in the *Foxn1* ^Δ/Δ^ periphery might be driven by an autoimmune response.

While DCs are the major stromal cells that perform negative selection at the cortical- medullary junction (CMJ) and in the medulla, TECs can also contribute to this process [9]. In this study, although the percentage of bone marrow derived DCs in the *Foxn1^Δ/Δ^* thymus was relatively increased, the capability of negative selection was still lower in the mice compared to in the *Foxn1* ^+/Δ^ mice. This reduction of negative selection may be due to the abnormal thymic architecture, with lack of both CMJ and medulla in the *Foxn1^Δ/Δ^* thymus, making it difficult for the SP thymocytes to interact properly with DCs. In addition, the reduction in negative selection could be secondary to premature exit of immature T cells from the thymus. In the normal thymus, SP thymocytes undergo maturation in the medulla for 10–14 days, during which time the expression level of HSA is down-regulated, and Qa2 is gradually up-regulated [24], [30], [31]. We found that the SP thymocytes and splenocytes expressed a higher percentage of HSA^hi^ and lower percentage of Qa2^+^ in newborn and day 14 *Foxn1^Δ/Δ^* mice. Consistent with the immature phenotype of *Foxn1^Δ/Δ^* peripheral T cells, some CD4^+^ T cells couldn't completely be activated under anti CD3 stimulation in vitro, although the CD8^+^ T cells appeared more normal than CD4 T cells (Fig. 5D). Other work in our lab has shown that the thymic vasculature in *Foxn1* ^Δ/Δ^ mice does not form properly, including inability to retain FITC-dextran (JL Bryson, et al., in preparation). This functional deficit combined with the lack of an organized medulla could combine to result in HSA^hi^ immature cells migrating out of the thymus prematurely in *Foxn1^Δ/Δ^* mice. Thus, the *Foxn1^Δ/Δ^* thymus is both inefficient at generating mature SP cells and unable to retain the resulting immature cells in the thymus, resulting in immature and autoreactive T cells in the periphery.

Positive selection of CD4 TCRαβ thymocytes is completely dependent on MHC II expressing TEC, while the development of CD8 thymocytes can be supported by MHC I on both TEC and other thymic stromal cells in thymus [32], [33], [34]. In *Foxn1^Δ/Δ^* thymus, the positive selection for CD4 transgenic T cells was dramatically damaged, while the selection for CD8 transgenic T cells was less affected (Fig. 3, 4). This difference between CD4 and CD8 is consistent with the greatly reduced expression of MHC II on TECs, while MHC I was almost normal in the *Foxn1^Δ/Δ^* thymus [10], (Fig. 5). This result was also consistent with our data from another *Foxn1* mutant, *Foxn^lacz^*, in which *Foxn1* expression is gradually down-regulated after birth. In these mice, MHC II expression on TECs and production of CD4 T cells was reduced, but CD8 T cells appeared normal [18]. The CD1 molecule was reported to support CD4 positive selection in MHC II^−/−^ mice [35]. CD1 expression was normal in *Foxn1^Δ/Δ^* thymus (data not shown), suggesting that it might support some positive selection for CD4 T cells in *Foxn1^Δ/Δ^* mice. Alternatively, the reduced positive selection could be due in part to an inability of the thymocytes themselves to respond to selection signals, either due to prior developmental deficits, or to intrinsic qualities based on their differentiation from an atypical progenitor cell [20].

These intrathymic defects in T cell differentiation and selection gave rise to peripheral T cells with defects in *in vivo* antigen-specific response. The antigen-specific response *in vivo* is a complicated process. Multiple facts may affect the process such as the dose of antigen, preparation of antigen, usage of adjuvant, the way of giving antigen, animal age and immune status [26], [27], [36], [37], [38], [39]. For example, oral feeding of antigen may result in tolerance through anergy [39]; intravenous injection of antigen may delete antigen specific T cells rapidly, with remaining cells hyporesponsive; while the subcutaneous injection of antigen may delete antigen specific T cells, with the remain cells hypersensitive [36]. Both low and high dose antigen can result in tolerance, but low dose of antigen results in anergy or clone deletion, while high dose of antigen results in active suppression of antigen specific T cells [26], [27]. During antigen specific tolerance, the CD25^+^CD4^+^ T regulatory cells that develop in the thymus play an important role in the process of tolerance and immune balance [38], [40]. In *Foxn1^Δ/Δ^* mice, the peripheral T cells had an abnormal response to specific antigens; CD4 T cells showed a delayed and strong response to SEA, while CD8 T cells had a delayed but weaker response compared to the same cells in the *Foxn1* ^+/Δ^ mice (Fig. 7), or to that reported in wild-type mice [41].

Both intrathymic and peripheral defects could contribute to the abnormal antigen response of *Foxn1* ^Δ/Δ^ T cells: 1) the deficient thymic environment, including the down-regulation of MHC II on TEC, might preferentially select the higher avidity T cells, and these cells could not be deleted by negative selection; 2) the abnormally lymphopenic peripheral environment causes lymphopenia induced proliferation (LIP) [20]; this could cause some clones with a higher avidity to be advantageously expanded in the *Foxn1* ^Δ/Δ^ periphery; 3) since CD4^+^CD25^+^ regulatory T cells played a key role in antigen specific tolerance in vivo [38], [40], [42], some phenotypes could result from the reduced function of CD4^+^CD25^+^ regulatory T cells in the *Foxn1* ^Δ/Δ^ mice [20]; and 4) constitutively low TCR expression on *Foxn1* ^Δ/Δ^ T cells may reduce the response to antigen [21]. The mutant-like response to SEA of wild-type or OT-1 transgenic SP T cells transferred in *Foxn1^Δ/Δ^* mice (Fig. 7) implicates the peripheral environment, rather than the development in an abnormal thymic microenvironment, in this abnormal response. Furthermore, we showed that wild-type CD4^+^CD25^+^ regulatory T cells transferred into *Foxn1^Δ/Δ^* mice could partially inhibit the strong response (Fig. 8E). Taken together, our results suggest that that the *Foxn1^Δ/Δ^* lymphopenic environment and the lack of normal function of CD4^+^CD25^+^ regulatory T cells were the major reasons for the hypersensitive response in the *Foxn1^Δ/Δ^* mice.

Taken together, our results show that both deficient development of T cells in the thymus and an abnormal antigen specific response in the periphery contribute to the phenotype of peripheral T cells in *Foxn1^Δ/Δ^* mice. Further analysis on this experimental system may be useful for the studies of clinical immune tolerance or vaccine immune response in humans with immune deficient disease.

## Materials and Methods

### Mice and treatment

*Foxn1^Δ/Δ^* mutant mice generated in our laboratory were described previously [10]. The mice were of a mixed C57BL/6J and 129SvJ background. BL/6-Ly5.1 mice, BALB/c mice, DO11.10 and OT1 TCR-transgenic mice were obtained from Jackson Labs. All transgenic strains were routinely genotyped by PCR. *Foxn1* ^Δ/Δ^;BALB/c mice were generated by backcrossing *Foxn1^Δ/Δ^* mice (H-2^b^, IE^-^) with BALB/c (H-2^d^, IE^+^) for more than 5 generations. To control for the H-2^d^ background, a pair of primers that are linkage marker D17MIT22 for H-2 IA and IE genes were used for genotypic PCR analysis. This primer pair produces a 157 bp fragment for the C57BL/6 allele, and a 183 bp-sized fragment from BALB/c allele. *Foxn1* ^Δ/Δ^;DO11.10 Tg and *Foxn1* ^Δ/Δ^;OT1 Tg mice were generated by mating *Foxn1* ^Δ/Δ^;BALB/c mice with DO11.10 mice and *Foxn1^Δ/Δ^* mice with OT1 mice, respectively. For testing the response of Vβ T cell clones to superantigen, mice were retro-orbitally injected with a single dose of 1 µg SEA (Staphylococcal Enterotoxin A, Sigma-Aldrich) per mouse, in 200 µl PBS. For response of OT1 Tg CD8 cells to OVA protein (Sigma, Steinheim, Germany, MW:44.287 Da), mice received single retro-orbital injections of a serial dose range from 0.06 to 20 mg in 200 µl PBS. All the mice were maintained in a specific pathogen-free (SPF) facility at University of Georgia; experiments were approved by the University of Georgia's Institutional Animal Care and Use Committee. The internal IACUC approval number currently is A2008-10181.

### Flow Cytometry Analysis and Antibodies

Splenocyte and thymocyte cell suspensions were prepared and counted for total cell numbers. Blood samples were taken by retro-orbital bleed on the indicated day; 50–100 µl blood was collected and blood mononuclear cells were isolated by density gradient centrifugation and lysis of RBC. All blood mononuclear cells and 0.5–1×10^6^ thymocytes or splenocytes were stained with the following mAbs conjugated to PE, FITC, allophycocyanin directly, or Biotin-labeled mAbs, followed by Strepavidin-PerCP: Mouse TCR Vβ screening panel, anti-CD4 (RM4-5), anti-CD8a (53-6.7), anti-CD45.1 (A20), anti-CD45.2 (104), anti-CD3e (145-2c11), anti-HSA (M1/69), and anti-Qa2 (H-2), anti-Kj1-26, anti-Vβ3 (Kj25), anti-Vβ5.1,5.2 (MR9-4), anti-Vβ11 (RR3-15), and anti-Vα2 (B20.1) anti-CD44 (IM7), anti-CD25 (7D4), anti-CD69 (H1.2F3), (all Abs from BD Pharmingen, San Diego, CA).

For the staining of MHCI and MHCII on CD45^-^ thymic stromal cells (TSC), thymic lobes were cut and washed in RPMI 1640+2%FBS medium to deplete most thymocytes, thymic fragments were then digested by collagenase/dispase (1 mg/ml, Roche Applied Science) plus DNase I (5 µg/ml) in RPMI 1640 plus 2%FBS medium for 1–1.5 hours at 37°C bath. The enriched TSCs were then stained with anti-CD45-allophycocyamin (30-F11), anti-MHCI H-2Kb PE (AF6-88.5), and MHCII I-A/I-E FITC (M5/114.15.2) (Biolegend). Anti-CD16/32 (2.4G2) (Biolegend or BD Pharmingen) and Rat-serum were used to block FC-receptors before staining. Three- or four-color immunofluorescence analysis was performed using a FACSCalibur system. The data were analyzed using CellQuest software (Becton Dickson, Franklin Lake, NJ).

### Activation of peripheral T cells by anti-CD3 antibody *in vitro*

2×106 peripheral lymphocytes prepared from spleen and LNs were plated in 24-well plates previously coated with 10 µg/ml of anti-CD3ε Ab and cultured for 24 h. As a control, cells were cultured in media alone. Cells were harvested after 9 and 24 hour culture and stained with anti-CD4, anti-CD8, and anti-CD44, anti-25 and anti-CD69 markers, then placed in FACS store solution for analysis by flow cytometry.

### Adoptive transfer

Peripheral lymphocyte suspensions were prepared from freshly isolated spleen and lymph nodes from C57BL6/J Ly5.1 or OT1 donor mice and further isolated by density gradient centrifugation and lysis of RBC in ACK lysis buffer (Cambrex Bio Science, Walkersville, MD). Around 4×10^6^ cells were transferred into *Foxn1^Δ/Δ^* mice. For isolation of T regulatory cells, peripheral lymphocyte suspension from *Foxn1* ^+/Δ^ mice was stained by anti-CD4 PE and anti-CD25 allophycocyanin mAbs, then sorted by MoFlo FACS (DakoCytomation). 2×10^5^ T regulatory cells per mice were transferred into *Foxn1^Δ/Δ^* mice.
